# Evaluation of heavy metals content in dietary supplements in Lebanon

**DOI:** 10.1186/1752-153X-7-10

**Published:** 2013-01-18

**Authors:** Samira Ibrahim Korfali, Tamer Hawi, Mohamad Mroueh

**Affiliations:** 1 Lebanese American University, P.O.Box 13–5053, Chouran Beirut, 1102 2801, Beirut, Lebanon; 2 Lebanese American University, P.O. Box 36, Byblos, Lebanon

**Keywords:** Dietary supplements, Heavy metals, Daily intake, Health/risk factor, XRF, Lebanon

## Abstract

**Background:**

The consumption of dietary supplements is widely spread and on the rise. These dietary supplements are generally used without prescriptions, proper counseling or any awareness of their health risk. The current study aimed at analyzing the metals in 33 samples of imported dietary supplements highly consumed by the Lebanese population, using 3 different techniques, to ensure the safety and increase the awareness of the citizen to benefit from these dietary supplements.

**Results:**

Some samples had levels of metals above their maximum allowable levels (Fe: 24%, Zn: 33%, Mn: 27%, Se: 15%, Mo: 12% of samples), but did not pose any health risk because they were below permitted daily exposure limit and recommended daily allowance except for Fe in 6% of the samples. On the other hand, 34% of the samples had Cu levels above allowable limit where 18% of them were above their permitted daily exposure and recommended daily allowance. In contrast, all samples had concentration of Cr, Hg, and Pb below allowable limits and daily exposure. Whereas, 30% of analyzed samples had levels of Cd above allowable levels, and were statistically correlated with Ca, and Zn essential minerals. Similarly 62% of the samples had levels of As above allowable limits and As levels were associated with Fe and Mn essential minerals.

**Conclusion:**

Dietary supplements consumed as essential nutrients for their Ca, Zn, Fe and Mn content should be monitored for toxic metal levels due to their natural geochemical association with these essential metals to provide citizens the safe allowable amounts.

## Background

Over the years the pharmaceutical industry has been one of the most stable industries in the world, manufacturing a wide range of therapeutic agents and dietary supplements. While the use of therapeutic agents and prescribed medications are considered essential to resist against many diseases [1]; dietary supplements are consumed to extend our diet with needed vitamins, minerals, herbs and amino acids for optimal body function [2,3].

Nowadays, the utilization of multivitamins or multiminerals preparations is widely spread to increase the daily intake of essential micronutrient. In the Unites States, it has been estimated that approximately 40% of the population consumes vitamins and/or mineral preparations [4]. Calcium, iron, manganese, zinc and copper are among these elements and are of great importance to our daily body functions due to their physiological and biological roles. The essentiality of Cu and Zn is based on its role as cofactor of a large number of enzymes [3-5]. Manganese is also a vital mineral, needed for normal growth; it helps breakdown fats, carbohydrates and proteins, and serves as co-factor for enzymes [5]. However, an increase in intake above recommended limit and daily allowance (RDA) for Mn (2–5 mg/day), Zn (8 mg/day female and 11 mg/day male) and Cu (1.5-3 mg/day) may result in toxic effects [3,5,6]. A high supplement of Cu has been related with liver damage and overdoses of Zn may weaken the immune system, reduce HDL levels (good cholesterol) and induce seizure [3-5]. High levels of Mn can pose a neurotoxic threat and chronic intake of high doses can cause Parkinson’s like symptoms [5]. Although the mechanism of action of Cr in the body and the amounts needed for optimal health care are not well defined, yet it is required in trace amounts. The biologically active form Cr^3+^ is believed to enhance the action of insulin [7], while Cr^6+^ is toxic and results from industrial pollution. Few serious adverse effects have been linked to high intake of Cr, and WHO [8] considered that supplements with chromium should not exceed 250 μg/day. But the recommended daily allowance (RDA) set by the Food and Drug Administration (FDA) is 35 μg/day [6]. Mo and Se are also essential minerals. Molybdenum is a component of important enzyme systems [2], and Se is incorporated into proteins producing important antioxidant enzymes the selenoproteins. The adverse health effects of selenium or selenium toxicity (selenosis) are hair and nail brittleness and other symptoms such as gastrointestinal disturbances, fatigue and irritability [9,10]. The recommended daily allowance (RDA) according to the Institute of Medicine (IOM) is 55 μg/day [11].

On the other hand, heavy metals such as Pb, Hg, Cd, and As are toxic at much lower levels. Lead is known to induce renal tumors, reduce cognitive development, increase blood pressure and cardiovascular diseases in adults. The human brain is most affected by lead intake. Children appear to be especially sensitive to lead, and lead exposure has been correlated to decreased IQ and poor learning in children [2,3,12-14]. Organic mercury is more toxic than inorganic form since it is more readily absorbed through ingestion; it is very harmful to fetal and children developments [15]. However, high exposure to organic and inorganic mercury may cause neurological disorders including seizures and even death [3]. Cadmium excessive intake affects mostly the kidney and to a lower extent the reproductive system [3], while that of arsenic is known to cause cancer [16], impairment of the reproductive system [17], and atherosclerosis [18]. The US Pharmacopeia [19] in its latest revision of metal limit has set the oral permitted daily exposure (PDE) from drugs and dietary supplements to be for: Pb 5 μg/day, Hg 15 μg/day, Cd 25 μg/day, and As 1.5 μg/day (previously set at 15 μg/day by USP in 2008 [20]).

The manufacturing of medicinal products requires extensive quality control, including the control of all manufacturing phases until the final product. Some countries have set strict quality control regulations and many others failed [21]. Several regulatory agencies highlighted that some dietary supplements may induce health problems with regard to their quality, effectiveness and safety for human consumption [22]. Poor quality control increases the risk of contamination of these products by bacteria, fungi, heavy metals and metalloids [21,23].

Many analytical techniques have been developed to determine different methods of metal concentration. Tumir et al. [21] used microwave digestion and Atomic Absorption Spectrometry to find the concentration of Pb, Cd, As, Hg, Cr, Ni, and Zn. Kaufman et al. [12] determined lead content of dietary supplements by ICP-MS. The approved FDA method is the ICP. However, a proposed protocol is set to acknowledge the recent EDXRF technique (Energy dispersive XRF, using X-ray tubes as an excitation source) if the samples have toxic metal concentrations greater than 10 ppm [24]. Better detection limits and more elements would be obtained using X-ray tubes as excitation sources instead of radionuclide sources. With modern software’s, it is possible to produce clean spectra with net peak intensities, and precise corrections for inter-element matrix effects [25]. Though literature cites limited use of XRF for determining metals in dietary tablets, yet XRF has been widely used for applications in metal analysis of alloys, soils, Pb in paints and Cd in plastics. It has been used for monitoring trace metals in milk [26], metals in oysters [27], trace metals in fruit juices [28], metals in oriental spices [29]. Additionally, Anderson [30], an FDA researcher, used hand-held XRF analyzer for determining toxic elements in tableware. While Chuparina and Aisueva [31] determined heavy metals in medicinal plants using XRF, as well as Bueno and Romão [32] determined P, Ca, Ti and Fe in counterfeit medicines.

Lebanese are becoming more health conscious and nutritional supplements have become a vital part of their daily diet, which led to a growing demand for dietary supplements including vitamins and minerals. This increase is due to the consumers’ beliefs that these products are natural and safe and devoid of any adverse effects. Unfortunately, this trust made most consumers use these dietary supplements without proper counseling and monitoring. Regulatory agencies in Lebanon highlighted the problems with dietary supplements in terms of quality, effectiveness and safety. However, these regulations are not implemented and not strictly enforced. Thus the objective of the current study is to evaluate metal content and metal contamination in dietary supplements available in Lebanese market (imported goods) using XRF technique and digestion/AAS technique, and ensure the safety and awareness of the citizens regarding these dietary supplements.

## Results and discussion

### Dietary samples characteristics

Thirty three dietary supplement tablets of Vitamin, Vitamins and Minerals, Minerals, and Herbs were purchased from local pharmacies. The brands were selected based on two criteria: first that they contain either metals only and/or metals as their main constituent, second for their popularity among the Lebanese citizens. The samples were coded according to their main dietary supplements: Vitamins were coded as V (3 samples), Vitamins and Minerals as VM (13 samples), Minerals as M (7 samples), and Herbs as H (10 samples). Table 1 represents the manufacturing country, usage and content as indicated on each Label.

**Table 1:** Characteristics of dietary tablets

**Sample ID**	**Origin**	**Therapeutic indication**	**Main ingredients**
Vitamins			
V1	Italy	healthy shiny hair	Niacin,Folic Acid, Vitamin B-12, Silica,
V2	USA	Improve microcirculation	Vitamin C, E, Creatine, Ginseng cert
V3	Belgium	physical and mental fatigue	Vitamin A, B6, B12, C, Ca, P, Zn, Cr
Vitamins and Minerals			
VM1	UK	suitable for diabetics	Vitamin A,B1,B6, Folic acid, Fe, Zn, Mn, Se, Cu
VM2	USA	essential nutrients and anti-oxidants	Vitamin D, Ca
VM3	Denmark	Multivitamins and minerals for children	Vitamin A, B1,B12,D,E,Cu,Fe, Mn, Mo, Se, Zn,
VM4	Italy	13 vitamins and 13 minerals	Vitamin A, B1,B5, B6,B12,C,D,E, PP, Ca, Fe, Cr, Cu, Fe, K, Mn, Mo, Se, Zn
VM5	USA	bones and teeth, prevent osteoporosis	Vitamin D3, Ca, magnesium silicate, mineral oil
VM6	UK	Multivitamins and minerals for adults 50	Multi-Vitamins,Ca, Cu, Fe,K, Ginkgo Biloba
VM7	USA	Energizer multivitamin	Multi-Vitamins, Ca, Cu, Fe,Mo, B, K,, Ginseng, Ginkgo Biloba, Green Tea, Spinach,
VM8	Switzerland	antioxidant effect, vitamin and mineral for vision and the eye	Beta-carotene, Vitamin B2, C, E, Cu, Se,Zn, Lutein
VM9	USA	supports heart health	Multi-Vitamins,Ca, Cu, Fe,Mo, B, K,P, V,Se,Ti,
VM10	UK	Mineral and Vitamins for pregnant women	Multi-Vitamins, Betacarotene, Cu,Fe, Zn
VM11	USA	Vitamin, mineral, herb formula for women	Multi-Vitamins, Ca, Cu, Fe, B, K,P, Se
VM12	USA	Vitamin, mineral, herb formula for men	Multi-Vitamins Ca, Cu, K, Mn, Se, Zn
VM13	USA	Vitamins and mineral formula for pregnant and lactating women	Vitamin A,B1, B2,B6,B12,C,D,Folic acid, Ca, Mg, Zn,
Minerals			
M1	USA	Calcium and Magnesium supplement	Ca- from calcium carbonate and oyster shells
M2	USA	bones and help prevent osteoporosis	Ca, Mg, Zn, eggshells and mineral oil
M3	USA	Calcium supplement	Ca, Mg, Cu, Mn
M4	Italy	Food supplement with fossil coral	Ca, Mg, Fossil coral powder
M5	UK	Helps maintain energy levels	Ca, Mg, Zn
M6	USA	Promotes sleep, and calm mental and stress	Ca, Mg, Valerian
M7	USA	Milk and dairy digestant	Lactase Enzyme,Soybean Oil
Herbs			
H1	USA	Memory Disorder	Ginkgo Biloba (dried Leaf and powder)
H2	USA	Digestive function	Pancreatin, Protease (Papain), Papaya Fruit Powder
H3	UK	Immune system	Stearic acid, beta-carotene selenium yeast
H4	USA	Immune function	Vegetable Stearic acid,and magnesium stearate,
H5	Italy	Urinary ducts	Proanthocyandian, bearberry and ortosiphon leaves
H6	Italy	Llight and moderate state of depression	L-Tryptophan, Vitamin PP, L-Tyrosine, L-Phenylalaninr, Vitamin B6
H7	USA	Support joint lubrication,	Hydrolized chicken sternal cartilage, Hyaluronic acid
H8	Italy	metabolic process	Ginkgo Biloba leaves
H9	USA	Memory Disorder	Ginkgo Biloba dried leaf and extract
H10	Canada	Aids in digestion	Amylase, bromelain, papain, papaya

### Concentrations of micronutrients with a high upper intake limit

The most abundant elements in the dietary samples were iron and zinc, followed by Mn. These metals are classified by USP stimuli article “General Chapter on Inorganic Impurities: heavy metals, Pharmacopeia form 34/5” as class IV that is recognized as micronutrient with a high upper intake limit [33]. Table 2 presents the concentration of Fe, Zn and Mn in studied samples and the maximum allowable levels (MAL) proposed by the Expert Committee in USP Advisory Panel on Inorganic Impurities and Heavy metals [34]. It also includes the recommended daily allowance (RDA) set by the Food and Drug Administration (FDA) [6], the permitted daily exposure (PDE) recommended by US Pharmacopeia, and permissible daily intake set by WHO/FAO calculated using provisional tolerable intake for a body weight of 60 kg and allocating 10% of this to be due to dietary supplement as indicated in Dietary Supplement –Standard 173 [35].

**Table 2:** Concentration of micronutrients with a high upper intake limit (Fe, Zn, Mn) in tablets compared to maximum accepted level, and daily intake (DI) as recommended on tablet label and compared to recommended daily allowance and permitted levels

**Sample ID**	**Fe (μg/g)**	DI* (μg/day) Fe	**Zn (μg/g)**	DI* (μg/day) Zn	**Mn (μg/g)**	DI* (μg/day) Mn
V1	1120	3841	3570	12245	328	1124
V2	346	252	14	10	5.5	4
V3	1836	2295	396	494	2.5	3
VM1	9439	6513	9986	6890	2028	1399
VM2	7659	12637	1918	3164	8.7	1396
VM3	5801	5743	1199	1187	743	735
VM4	11788	5894	5108	3554	1962	981
VM5	36	55	3.5	5	17	25
VM6	2420	4654	1839	2776	10	15
VM7	9389	15117	2784	4482	1409	2269
VM8	72	97	16054	21673	3.5	5
VM9	41	62	3136	4705	852	1278
VM10	21280	15534	9895	6566	132	96
VM11	1942	8465	823	3586	361	1575
VM12	35	128	1307	4731	639	2315
VM13	13494	19162	5067	7195	43	62
M1	82	253	2.1	7	63	196
M2	24	31	961	1250	4.4	5.2
M3	56	212	1263	4799	480	1826
M4	58	186	3.5	11	14	45
M5	6	24	806	3553	6.5	29
M6	68	133	6.7	13	13	25
M7	25	70	6.1	8	8.2	11
H1	578	399	3.3	3	12	8
H2	50	65	1.1	1.4	1.1	0.7
H3	888	462	10.5	5.5	1.0	0.5
H4	5	3	4452	2894	1.5	0.9
H5	85	66	8.4	6.5	59	46
H6	47	34	2.0	1.5	4.5	3
H7	26	19	10.5	7.7	5.3	4
H8	47	25	2.9	1.6	6.2	3
H9	15	5	1.0	0.3	2.1	0.6
H10	27	14	1.8	0.9	3.3	1.5
MAL**	1500^a^	-	1500^a^	-	250^b^	
PDE^†^	-	15000 ^a^	-	15000 ^a^		2500 ^b^
RDA/FDA^‡^	-	8000 ^c^	-	8000 ^c^ (female)	-	-
				11000 ^c^ (male)		
WHO/FAO^‡‡^	-	4800 ^d^	-	6000 ^e^	-	-

*DI: daily intake calculated from mass of tablet and labeled recommended tablets per day.

**MAL: Maximum accepted levels of metals in Tablets (μg/g)according to US Pharmacopeia (USP).

^†^PDE: Permitted Daily exposure (μg/day) according to US Pharmacopeia (USP).

^‡^RDA: Recommended daily allowance after FDA [6].

^‡‡^WHO/FAO : Permissible daily intake calculated from using provisional tolerable intake and body weight of 60 kg and 10% due to dietary supplements [35].

^a^USP [20]^b^ USP [34]^c^ FDA [6]^d^ JECFA [36]^e^ JECFA [38].

The mean levels for Fe, Zn and Mn were 2690, 2133 and 305 μg/g, respectively. The highest levels of these metals were in Vitamins and Minerals with respectively mean levels of 6145, 4478 and 696μg/g, and were above their respective maximum allowable level (MAL) of 1500, 1500 and 250 μg/g [34]. Additionally, 7 (45%) of the Vitamins and Mineral samples had levels of these metals above their recommended MAL. For example, VM10, had the highest level of Fe (21280 μg/g), 14 times more than it’s MAL, and this product is advised as a supplement during pregnancy. The daily intake of iron from this product is 15,534 μg/day, and it slightly exceeds the maximum PDE (15000 μg/day), and is twice the FDA recommended daily allowance of 8000 μg/day. Similarly, VM13, an advised vitamin and mineral formula for pregnancy and lactation, had a high level of Fe (13, 494 μg/g) and its daily recommended intake is 19161 μg/day (1.3 times its PDE, and 2.3 times more than its RDA), while VM4 had Fe level of 11789 μg/g, but its daily intake (5894 μg/day) is below the recommended RDA and PDE levels. But compared to permissible daily intake by WHO/FAO [36] from provisional tolerable daily intake of 0.8 mg/kg body weight, for 60 kg and allocating 10% to dietary sample [35], then 24% of samples are above permissible levels. Nevertheless according to the National Institute of Health, Office of Dietary Supplements [1], the recommended daily allowance (RDA) for women is 18 mg/day, 27 mg/day during pregnancy and 10 mg/day for lactating women. Both products with high Fe content (VM10 and VM13) are recommended during pregnancy and lactation and do not pose any health concern in terms of Fe levels, because it is still below Tolerable Upper Intake levels (45,000 μg/day).

The highest level of Zn was found in VM8 (16054 μg/g) which is about 10 times more than its recommended maximum allowable level-MAL (1500 μg/g) and its daily intake (21673 μg/day) exceeds the set PDE (15000 μg/day), and is twice the set RDA value (Table 2). This product labeled as antioxidant, contains minerals and vitamins needed for healthy vision. Moreover 33% of all samples and 61% of Vitamin and Minerals samples had levels of Zn above their recommended MALs. Though Zn is an essential constituent of many enzymes, needed for protein synthesis, and energy metabolism, yet excess intake may lead to electrolyte imbalance and nausea [37]. The Office of Dietary Supplements [1] of the National Institute of Health set a daily requirement for zinc between 8 – 12 mg/day for adults. As such, the highest daily intake of Zn in sample MV8 exceeds the RDA value, but is still below Tolerable Upper Intake levels (40 mg/day), and hence there is no health risk of Zn intake in all these samples. Also 12% of samples exceeded WHO permissible limits based on provisional tolerable daily intake for 60 kg body weight and 10% allocation to dietary supplements [35,38]. Compared to previous studies including multivitamin/mineral supplements, the reported Zn levels in this study were higher than those in dietary supplements in Mexican market (2.83- 4786 μg/g [3]; but were comparable to Avula et al. [39] with Zn ranges were between 170 and 22,500 μg/serving.

The mean level of Mn in all samples was 305 μg/g and is higher than its maximum allowable level (MAL). Similar to Fe and Zn, the Mn levels were highest in Vitamins and Minerals supplements, with a mean level of 696 μg/g. Mn content was very high in sample VM1 with a value of 2028 μg/g, exceeding by about 8 times the MAL (250 μg/g) set value. The therapeutic indication of VM1, as per label, is “essential vitamins and minerals needed for diabetics”. Nevertheless, the Mn daily intake in all samples was below their PDE set levels (2500 μg/day), and hence do not pose a health concern. Literature review shows that the reported Mn levels in multivitamin/mineral supplements are within the studied range as reported by Avula et al. [39] with an Mn range of 78 and 17,500 μg/serving.

The three metals showed a statistical significant correlation among studied samples (for Fe and Zn, r = 0.672, p < 0.01; Fe and Mn r = 0.554, p < 0.01, and Zn and Mn r = 0.518, p < 0.01). Thus either these minerals are added each as a supplement and/or they are geochemically associated to each other. Iron and Mn belong to the same class being considered geochemically as reducible fractions forms, and Zn is highly adsorbed and associated with these reducible fractions [40-42].

### Concentrations of micronutrients with low established upper intake limit

Copper, Cr, Mo, and Se metals are classified by USP stimuli article “General Chapter on Inorganic Impurities: Heavy metals, Pharmacopeia form 34/5” as class III and are recognized as micronutrient with a low established upper intake limit [33]. Hence, these elements are regarded essential nutrients with trace amounts, but become toxic at high concentrations. Table 3 presents the concentration of Cu, Cr, Mo, and Se of studied samples, the maximum allowable levels (MAL), the recommended daily allowance (RDA), the permitted daily exposure (PDE), and the permissible daily intake of respective metals from dietary supplement.

**Table 3:** Concentration of micronutrients with low established upper intake limit (Cu, Cr, Mo, Se) in tablets compared to maximum accepted level, and daily intake (DI) as recommended on tablet label and compared to recommended daily allowance and permitted levels

**Sample ID**	**Cu (μg/g)**	DI* (μg/day) Cu	**Cr (μg/g)**	DI* (μg/day) Cr	**Mo (μg/g)**	DI* (μg/day) Mo	**Se (μg/g)**	DI* (μg/day) Se
V1	0.5	1.8	0.08	2.7	0.8	2.7	2.0	6.9
V2	9.0	6.6	0.04	0.03	3.4	2.4	0.1	0.08
V3	7.8	9.8	0.23	0.29	1.0	1.3	7.8	9.8
VM1	1583	1092	5.5	2.4	7.7	5.3	60.1	41.4
VM2	224	370	0.88	1.5	26	43	30.7	50.7
VM3	185	183	0.58	0.58	4.1	4.0	13.8	15.5
VM4	867	434	0.37	0.18	0.8	0.4	18.8	9.4
VM5	9.5	14.3	0.07	0.11	0.6	0.9	1.0	1.5
VM6	70	106	0.05	0.07	0.4	0.6	2.0	3.0
VM7	867	1396	0.31	0.49	35.4	57	24.9	40
VM8	1952	2635	0.02	0.03	13.3	18	52.9	71
VM9	725	1087	1.26	1.89	44.4	67	12.8	19
VM10	1270	927	0.17	0.12	0.5	0.4	0.9	0.7
VM11	225	979	0.54	2.30	0.6	2.6	7.3	32
VM12	281	1018	0.32	1.14	0.4	1.5	17.0	61
VM13	1.1	1.42	0.03	0.05	0.6	0.8	4.0	5.7
M1	6.9	22	0.06	0.20	0.7	2.2	2.1	6.2
M2	6.5	8.5	0.15	0.19	0.5	0.7	0.5	0.7
M3	376	1429	0.05	0.19	1.0	3.8	0.5	1.9
M4	7.9	25	0.13	0.41	0.3	1.0	1.1	3.2
M5	1.5	6.6	0.04	0.18	0.7	2.9	5.3	22
M6	8.8	17	0.10	0.19	0.8	1.6	2.2	3.9
M7	5.2	6.8	0.02	0.02	0.9	1.9	3.4	5.0
H1	8.5	5.8	0.08	0.05	1.1	0.7	1.1	0.7
H2	5.6	7.2	0.02	0.02	3.7	4.8	4.4	5.2
H3	3.8	2.0	0.03	0.02	0.5	0.3	200	104
H4	37	24	0.02	0.01	9.6	6.3	3.3	2.0
H5	15	12	0.09	0.07	7.5	5.8	3.1	2.3
H6	350	256	0.07	0.05	5.2	3.8	0.4	0.3
H7	16.5	12	0.02	0.01	6.5	4.7	0.3	0.2
H8	18.9	10	0.02	0.01	9.3	4.9	0.8	0.4
H9	5.8	1.8	0.07	0.02	0.1	0.03	4.1	1.3
H10	6.5	3.2	0.02	0.01	0.9	0.5	5.3	2.5
MAL**	100 ^a^		15^b^		10 ^a^		25^c^	
PDE^†^		1000 ^a^		150 ^b^		100 ^a^		250 ^c^
RDA/FDA^‡^		900 ^d^		35 ^d^		34 ^d^ (male) 45 ^d^ (female)		55 ^f^
WHO/FAO^‡‡^		300 ^e^		-		-		-

*DI: daily intake calculated from mass of tablet and labeled recommended tablets per day.

**MAL: Maximum accepted levels of metals in Tablets (μg/g) according to US Pharmacopeia (USP).

^†^PDE: Permitted Daily exposure (μg/day) according to US Pharmacopeia (USP).

^‡^RDA: Recommended daily allowance after FDA [6].

^‡‡^WHO/FAO : Permissible daily intake calculated from using provisional tolerable intake and body weight of 60 and 10% due to dietary supplements [35].

^a^USP [19]^b^ USP [34]^c^USP [20].

^d^ FDA [6]^e^ JECFA [38]^f^ IOM [11].

The mean level of Cu in all samples was 277 μg/g which is higher than its recommended MAL (100 μg/g), 34% (12 samples) of samples having concentrations above allowable levels. The highest Cu levels were in Vitamins and Mineral samples, with a mean of 635 μg/g, with 54% of these samples exceeding MAL values. The highest Cu level was found in VM 8 (1952 μg/g) and exceeds by 20 times the set MAL level. The indication of this product as per label is “vitamins and mineral salts necessary for healthy vision”. Similarly, VM1 had high Cu content (1583 μg/g) and is indicated as “essential vitamins and minerals for diabetics”. Two other samples, VM10 and VM7, had levels of 1270 and 867μg/g, and described as “essential vitamins and minerals for pregnant women”, and “essential energizer multivitamins”, respectively. The daily intake of Cu in VM 8, VM1 and VM7 are 2635, 1902 and 1396μg/day. All of these values are higher than the set PDE level of 1000 μg/day [19], and RDA levels of 900 μg/day [6], and WHO permitted daily intake of 300 μg/day [38] calculated from provisional tolerable daily intake for a body weight of 60 kg and allocating 10% due to dietary supplements. Consequently, these brands should be controlled and advised for their usage. Compared to other works of multivitamins and minerals, our studied Cu levels in samples were within the range of Cu in various multivitamins and multi-minerals (1 μg - 1981 μg per serving) reported by Raman et al. [2].

Unlike other metals, the Cr levels in the tested samples were low. The mean level in all samples was 0.29 μg/g and it was highest in Vitamins and Minerals (0.63 μg/g). The highest Cr level was 5.5 μg/g, thus 3 times less than its maximum allowable level (MAL) of 15 μg/g. Chromium levels were also below those reported by Avula et al. [39] for multimineral and multivitamin supplements with a range between 4 μg/serving and 248 μg/serving.

The calculated mean value of Mo in Vitamins and Minerals of 10 μg/g is identical to the maximum allowable level (MAL) Table 3[19]. Thirty percent of these samples had Mo levels higher than the MAL level, with the highest in sample VM9 of 44 μg/g. This sample is constituted of vitamins and minerals and recommended for adults (above 50) to support heart health. VM7, classified also as an energizer multivitamin, had a level of 35 μg/g. Nevertheless, the daily intake (μg/day) of Mo in all samples was below the permitted daily exposure limit (PDE) of 100 μg/day [19]. However, samples VM2 (43 μg/day), VM7 (57 μg/day), and VM9 (67 μg/day) exhibited daily intake above the recommended daily allowance (RDA) of 45 μg/day for males and 34 μg/day for females [6]. Thus these samples should be consumed with caution, not exceeding the recommended dose.

Though the mean level of Se (19 μg/g) is lower than the recommended set MAL (25 μg/g) value, yet four samples exhibited levels higher than 25 μg/g. The highest Se level of 200 μg/g was in H3 sample (herbs category) and indicated to maintain a healthy immune system. The three other samples were VM1, with a Se level of 60 μg/g, VM8 with 53 μg/g, and VM2 with 31 μg/g. Nevertheless all samples showed a daily intake level of Se below the permitted daily exposure (PDE) value of 250 μg/day [20]. However, considering the Se RDA value of 55 μg/day and its upper limit (UL) of 400 μg/day [11], then samples VM8 with 72 μg/day and H3 with 104 μg/day are above the recommended daily intake value levels but far below the upper limit [11]. Still, tablets of H3 should be consumed cautiously.

### Concentrations of toxic elements

Lead, Hg, Cd, and As are classified by USP stimuli article “General Chapter on Inorganic Impurities: Heavy metals, Pharmacopeial form 34/5” as class I. These are metal impurities and generally recognized as presenting significant toxicity [33]. Table 4 presents the concentrations of Pb, Hg, Cd, and As in studied samples, the maximum allowable levels (MAL), the permitted daily exposure (PDE), and the permissible daily intake of respective metal from dietary supplement. Since these metals are toxic, there are no recommended daily allowance levels.

**Table 4:** Concentration of toxic metals (Pb, Hg, Cd, As) in tablets compared to maximum accepted level, and daily intake (DI) as recommended on tablet label and compared to recommended daily allowance and permitted levels

**Sample ID**	**Pb (μg/g)**	DI* (μg/day) Pb	**Hg (μg/g)**	DI* (μg/day) Hg	**Cd (μg/g)**	DI* (μg/day) Cd	**As (μg/g)**	DI* (μg/day) As
V1	0.125	0.43	0.014	0.048	2.333	7.889	0.514	1.700
V2	0.161	0.12	0.015	0.011	0.552	0.401	0.095	0.069
V3	0.269	0.34	0.026	0.033	2.510	3.125	0.075	0.094
VM1	0.126	0.09	0.035	0.024	2.914	2.001	0.477	0.345
VM2	0.074	0.12	0.022	0.036	2.554	4.208	0.514	0.848
VM3	0.074	0.07	0.021	0.021	0.881	0.871	1.621	1.800
VM4	0.044	0.02	0.011	0.005	2.621	1.300	0.608	0.304
VM5	0.086	0.13	0.550	0.831	0.319	0.482	0.115	0.174
VM6	0.098	0.15	0.065	0.098	2.205	3.058	0.097	0.147
VM7	0.045	0.07	0.045	0.072	2.131	3.431	0.606	0.310
VM8	0.092	0.12	0.075	0.101	2.446	3.302	0.065	0.088
VM9	0.067	0.10	0.115	0.173	2.326	3.489	0.254	0.381
VM10	0.132	0.10	0.065	0.047	2.227	1.626	1.095	0.796
VM11	0.067	0.29	0.038	0.166	1.921	8.371	0.086	0.375
VM12	0.077	0.28	0.025	0.091	1.633	5.911	0.045	0.163
VM13	0.096	0.14	0.082	0.114	0.107	0.152	0.574	0.815
M1	0.071	0.22	0.093	0.278	0.821	2.537	0.013	0.038
M2	0.029	0.04	0.055	0.072	2.516	3.271	0.034	0.045
M3	0.071	0.27	0.044	0.167	1.820	6.916	0.057	0.217
M4	0.41	1.29	0.078	0.249	0.222	0.638	0.014	0.046
M5	0.012	0.05	0.015	0.066	2.139	9.433	0.068	0.300
M6	0.13	0.25	0.025	0.049	2.534	4.850	0.035	0.068
M7	0.097	0.13	0.044	0.054	0.196	0.143	0.066	0.089
H1	0.206	0.14	0.121	0.083	2.551	1.760	0.047	0.034
H2	0.109	0.14	0.255	0.329	1.493	1.922	0.055	0.071
H3	0.039	0.02	0.095	0.049	0.143	0.074	0.466	0.242
H4	0.247	0.16	0.085	0.055	0.540	0.351	0.699	0.454
H5	0.269	0.21	0.351	0.270	2.473	1.902	0.012	0.009
H6	0.029	0.02	0.243	0.175	0.294	0.288	0.023	0.017
H7	0.005	0.01	0.027	0.020	0.834	0.609	0.032	0.023
H8	0.045	0.02	0.011	0.006	0.599	0.317	0.076	0.040
H9	0.271	0.09	0.098	0.031	0.096	0.031	0.056	0.018
H10	0.058	0.03	0.011	0.005	0.086	0.042	0.022	0.011
MAL**	0.5 ^a^		1.5 ^a^		2.5 ^a^		0.15 ^a^	
PDE^†^		5 ^a^		15 ^a^		25 ^a^		1.5 ^a^
RDA/FDA^‡^								
WHO/FAO^‡‡^		21.4 ^b^		3.42 ^c^		6 ^d^		10 ^e^

*DI: daily intake calculated from mass of tablet and labeled recommended tablets per day.

**MAL: Maximum accepted levels of metals in Tablets (μg/g)according to US Pharmacopeia (USP).

^†^PDE: Permitted Daily exposure (μg/day) according to US Pharmacopeia (USP) for 50 kg body weight.

^‡^RDA: Recommended daily allowance after FDA [6].

^‡‡^WHO/FAO : Permissible daily intake calculated from using provisional tolerable intake and body weight of 60 kg and 10% due to dietary supplements [35].

^a^USP [19]^b^ JECFA [38]^c^ JECFA [43]^d^ JECFA [47]^e^ JECFA [50].

The levels of lead and mercury in all dietary samples were low. The mean levels of Pb and Hg in all samples were respectively 0.11 μg/g and 0.08 μg/g, with corresponding ranges of 0.01 – 0.41 μg/g, and 0.01 – 0.55 μg/g. Both Pb and Hg levels were lower than their maximum allowable levels (MAL) of 0.5 μg/g for Pb, and of 1.5 μg/g for Hg [19]. These levels were also way below the permissible daily intake calculated from provisional tolerable weekly intake (PTWI) of 25 μg/kg body weight for Pb [38], and of 4 μg/kg body weight for Hg [43], adjusted per day for a 60 kg body weight and allocating 10% for supplementary dietary tablets [35]. The levels of Hg (0.02 -0.12 μg/g) and Pb (0.25-3.86 μg/g), in dietary supplementary tablets of vitamins and minerals and herbs, reported by Tumir et al. [21] are compatible with the Hg levels in this study, yet their Pb levels are much higher.

**Figure 1:**
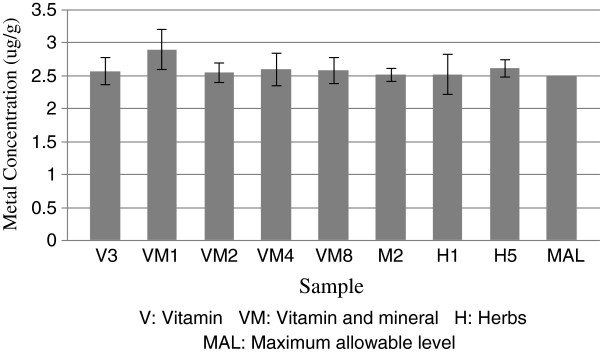
Samples above the maximum allowable Cd levels.

The mean level of Cd in all samples was found to be 1.8 μg/g, with a range of 0.08 to 2.9 μg/g. Eight of the samples had Cd levels above their maximum allowable level (MAL) of 2.5 μg/g as illustrated in Figure 1. Furthermore, 4 of the Vitamins and Minerals samples had levels above the recommended MAL. The highest level of Cd was found in sample VM1 (2.914 μg/g) followed by VM4 with a level of (2.621 μg/g). VM1 is labeled as “essential vitamins and minerals for diabetics”, while VM4 as “supplement multicomplex vitamins and minerals”. The other 4 samples with high Cd levels (above MAL) were from the other categories. For example the level in H5 sample was 2.62 μg/g and is recommended for the wellbeing of the urinary ducts, H1 of 2.551 μg/g and advised for memory disorder. The high levels of Cd in these samples resulted most probably from other essential minerals existing in the samples such as Zn, Ca and P. A statistical significant correlation existed between the levels of Cd and Zn (r = 0.75, p < 0.01), and most samples that had Ca and/or P as a constituent had high Cd levels. For instance, V3 (rich in calcium and phosphorous), H1 sample contains di-calcium phosphate, and H4 contains calcium phosphate. It is well established that Zn and Cd are geochemically related [41], and also Cd is an out product of phosphate rocks [44-46]. Cadmium and Ca have similar ionic radii and Cd can co-precipitates with calcium carbonate and/or has a high affinity for carbonates [40-42]. The daily intake of Cd in all samples, with a range of 0.03 - 9.4 μg/day is lower than the permitted daily exposure from drugs and dietary supplements (PDE of 25 μg/day). Yet 3 samples had higher levels than those calculated from FAO/WHO provisional tolerable intake [47], for a 60 kg body weight and 10% allocation due to dietary supplements (Table 4). The studied samples levels were below the MRL level (minimum risk level) of 14 μg/day [39]. MRL is an estimate of the daily exposure to a hazardous substance that is likely to be without appreciable risk of adverse non cancer health effects over a specified duration of exposure. Even if Cd levels were below referenced safety and health values, yet due to natural occurrences of Cd with essential metals (Ca, Zn), dietary supplements should be checked for content of Cd in samples that are essential for delivering the nutrients such as Ca and Zn.

**Figure 2:**
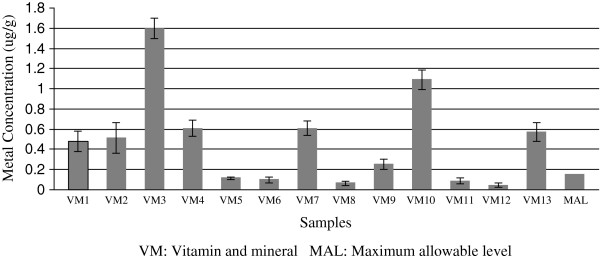
Levels of As in samples composed of vitamins and minerals.

**Figure 3:**
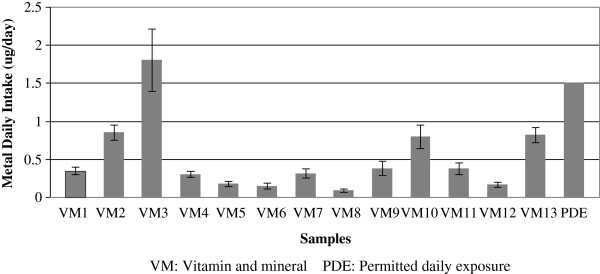
Levels of As daily exposure in samples composed of vitamins and minerals.

With respect to As, the mean level of all dietary samples was 0.26 μg/g, with a range between 0.012 and 0. 16 μg/g. Nine samples had levels above the newly revised MAL (0.15 μg/g) as set by US Pharmacopeial in 2012 [19]. Mineral and Vitamins samples were highest in As levels, and eight samples had levels above the recommended MAL (Figure 2). The highest As levels was in sample VM3 (1.621 μg/g), which is assigned as multi vitamins and minerals for children. VM10 As level was relatively high (1.095μg/g) and it is classified as “essential vitamins and minerals for pregnant women”. Whereas VM4 (labeled as “supplement of multi vitamins and minerals and does not result in accumulation when taken accordingly”) and VM7 (an energizer) had levels of 0.61 μg/g, and V1 had a level 0.514 μg/g. Furthermore a significant statistical correlation existed between the levels of As and those of Fe (r = 0.55, p < 0.05) and Mn (r = 0.67, p < 0.01). The geochemical association between As and Fe, As and Mn has been reported by Goh and Lim [48] and Kim et al. [49]. The mean daily intake of As for all samples was 0.3 μg/day, whereas for vitamin and mineral samples it was 0.5 μg/, with a range of 0.01 – 1.8 μg/day. Two samples had daily As intake values (Figure 3) higher than the oral permitted daily exposure (PDE of 1.5 μg/day) as revised by USP in 2012 [19]. But the As daily intake levels of all samples is lower than the previous level of 15 μg/day set by USP in 2008 [20], and lower than the recommended permissible daily intake by WHO/FAO [50] (Table 4). Furthermore, all samples had lower levels than the MRL level of 21 μg/day [39]. Yet even if the levels of As in all our samples were below the referenced values for safety and health, our dietary supplements should be monitored for content of As in dietary samples that have high Fe and Mn as essential nutrient.

## Conclusions

• Dietary supplements are generally utilized by most people on voluntary basis and without strict supervision and knowledge of their health/risk factor, in contrast to medications which are under control of Physicians. This project has evaluated the quality of 33 imported dietary supplements in terms of metals consumed by the Lebanese population.

• The sequence of the highest metals’ concentrations in all supplements was Fe, Zn, and Mn respectively.

• Statistical significant correlations occurred among the three metals (Fe, Zn, and Mn) indicating that either these minerals are added as supplements or as natural geochemical associations.

• With regard to the micro minerals, Cr concentrations were found to be less than the international maximum accepted levels. While in some samples, concentrations of Se and Mo were above the accepted limits, yet they were below the daily permitted exposure levels posing no health risk factor. On the other hand, some samples had levels of copper above allowable levels and permitted daily exposure, thus, it is recommended to monitor dietary supplements for their Cu levels.

• The health hazard effect level of the toxic metals Hg and Pb was absent in all studied samples.

• In contrast, 30% of the analyzed samples had levels of Cd above allowable levels, and were statistically correlated with Ca, and Zn essential minerals. 62% of samples had also levels of As above accepted limit and these levels were associated with Fe and Mn essential minerals.

• Consequently, supplements consumed as essential nutrient for their Ca, Zn, Fe and Mn should be monitored for toxic metal levels due to their natural geochemical association with essential metals to ensure the safety of supplement consumptions.

• However, exact justification of this association requires subjecting samples to chemical speciation analysis.

## Methods

### Reagents and glassware

All reagents used were of analytical reagent grade. High quality water, obtained using a Milli-Q system (Millipore, Direct Q equipped with ultrapure Ion-Ex Cartridge, C.No. QTUM000IX Bedford, MA, USA), was used exclusively).

All glassware was thoroughly cleaned by soaking overnight in 2 M nitric acid and then rinsing thoroughly with MilliQ water, then kept in an oven at 110°C.

### Sample preparation and analysis by EDXRF

The tablets of each sample and the content of each capsule were grinded, reduced to a fine homogenous powder, sieved using 2 mm plastic sieve size, and stored in plastic bags for metal analysis.

The concentrations of 6 metals (Ca, Cu, Fe, Mn, Se, Zn) in the prepared samples were determined using handheld XRF technique (Niton XL3 GOLLD hand held, Thermo Fisher Scientific) which is energy dispersive x-ray fluorescence (EDXRF) up to 50kV x-ray tube source and optimized silicon drift detector (SDD).

### Digestion procedures of samples

Samples of low metal concentrations were analyzed by the digestion method. The digestion procedure involved adding slowly and in portions 50 mL of freshly prepared 1:1 (v:v) H_2_O_2_ (30%): HNO_3_ (65%) to one gram of each sample, then covered and kept for 2 days. Each solution was heated till it became clear, then filtered into a 25 mL lidded plastic tube and diluted with deionized water, next inserted in a water bath at 60°C for 30 minutes, and stored at 4°C. Whereas, for samples that did not form clear solutions, 10 mL aqua regia was added before heating in oil bath at 120°C till clear, then filtered and stored similarly as above.

### Analysis by thermal AAS and ICP-MS

The concentrations of Cd, Cr, Mo, and Pb, in the prepared and digested dietary samples were determined by using AAS-Graphite furnace (“Shimadzu” AA-6300) and background correction deuterium lamp. Working standard solutions were prepared by dilution of stock solutions (1 mg metal/ml in 2% HNO_3_, Merick, Germany) with MilliQ water. The instrument detection limits were Cd: 2 μg/L, Cr: 4 μg/L; Pb: 3 μg/L, and Mo: 30 μg/L.

The concentrations of As and Hg were determined by ICP-MS (Agilent ICP-MS 7500-Ce, Japan). The instrument detection limits on the ICP-MS were 50 ppt for Hg, and 25 ppt for As.

### Reliability and efficiency of analytical techniques

Certified Reference Material (CRM) was used, including Tomato leaves (NIST 1573a) and soil (NIST 2586), to determine the efficiency and accuracy of the analytical methods and digestion procedures. Reference materials were done in triplicates. The recovery percentages based on CRMS for XRF technique are presented in Table 5, and those of metals analyzed by digestion in Table 6.

**Table 5:** Percent recovery of reference material by XRF (Expected Value)

	**Cu (μg/g)**	**Fe (μg/g)**	**Mn (μg/g)**	**Se (μg/)**	**Ti (μ/g)**	**Zn (μg/g)**
Tomato leave Certified (NIST 1573a)	4.7 ± 0.14	368 ± 7	246 ± 8	14.89 ± 0.27	-	30.9 ± 0.7
Measurement	4.42 ± 0.18	371 ± 9	242 ± 10	13.12 ± 0.31	-	31.2 ± 0.8
% Recovery	94	101	98	88	-	101
						
Soil Certified (NIST 2586)	-	51610 ± 890	1000 ± 18	-	6050 ± 660	352 ± 16
Measurement	-	48513 ± 700	903 ± 27	-	5627 ± 500	335 ± 20
% Recovery	-	94	90	-	93	95

**Table 6:** Percent recovery of reference material by digestion (Expected values)

	**As (μg/g)**	**Cd (μg/g)**	**Cr (μg/g)**	**Hg (μg/)**	**Pb (μ/g)**
Tomato leave Certified (NIST 1573a)	0.112 ± 0.004	1.52 ± 0.04	1.99 ± 0.06	0.034 ± 0.004	-
Measurement	0.111 ± 0.008	1.55 ± 0.06	1.95 ± 0.05	0.033 ± 0.006	-
% Recovery	99	101	98	97	-
					
Soil Certified (NIST 2586)	8.7 ± 1.5	2.71 ± 0.54	301 ± 45	0.367 ± 0.038	432 ± 17
Measurement	7.9 ± 2.1	2.49 ± 0.47	268 ± 27	0.342 ± 0.027	411 ± 24
% Recovery	91	92	89	93	95

## Abbreviations

DI: Daily intake; DIVM: Daily intake of vitamin and mineral; FDA: Food and drug administration; H: Herbs; MAL: Maximum allowable levels; M: Minerals; PDE: Permitted daily exposure; V: Vitamin; VM: Vitamins and minerals; USP: United states pharmacopeia; WHO: Worlds health organization.

## Competing interests

The authors declare no competing interests.

## Authors’ contributions

MM have recommended the project and have been involved in drafting the manuscript and revised it critically for soundness of the pharmaceutical concepts. SIK carried the project design, data analysis, interpretations, and prepared the manuscript. TH carried out all experimental procedures and acquisition of data. All authors read and approved the final manuscript.
